# Supplementary material for: Comparative survey of the relative impact of mRNA features on local ribosome profiling read density
Source: Nat Commun. 2016 Oct 4;7:12915. doi: 10.1038/ncomms12915 (PMC5059445; doi:10.1038/ncomms12915)
Supplement: Supplementary Information — Supplementary Figures 1-15 and Supplementary Table 1. [file ncomms12915-s1.pdf]

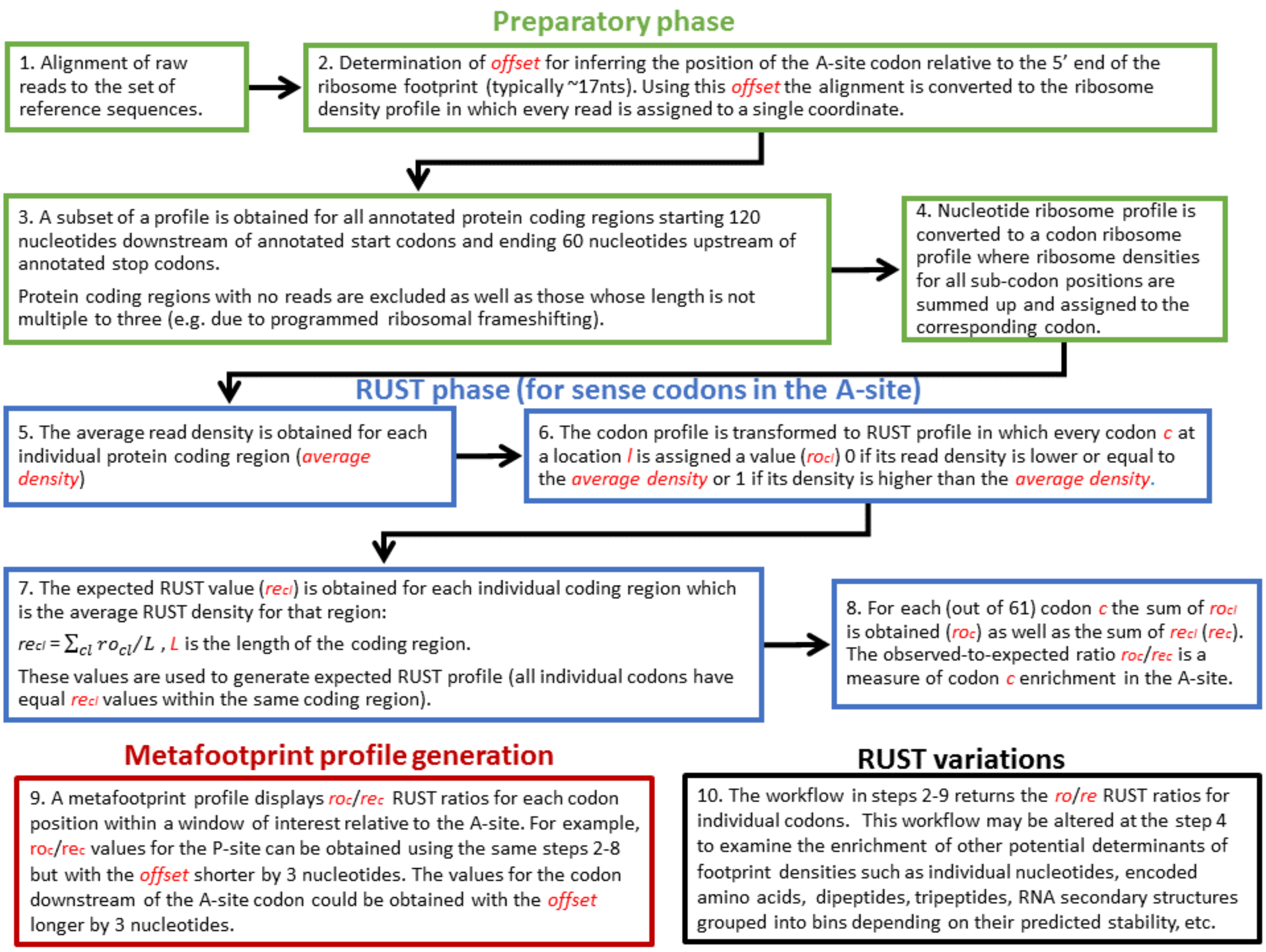

Supplementary Figure 1. RUST pipeline

Steps 2-8 describe the pipeline for the analysis of the codon enrichment at the ribosome A-site. Metafootprint box (bottom left) explains how the pipeline needs to be altered to obtain the data for other positions in mRNA (also see Supplementary Fig. 3 for more details) and RUST variations box (bottom right) explains how to alter the pipeline to analyse determinants of footprint density other than codons.

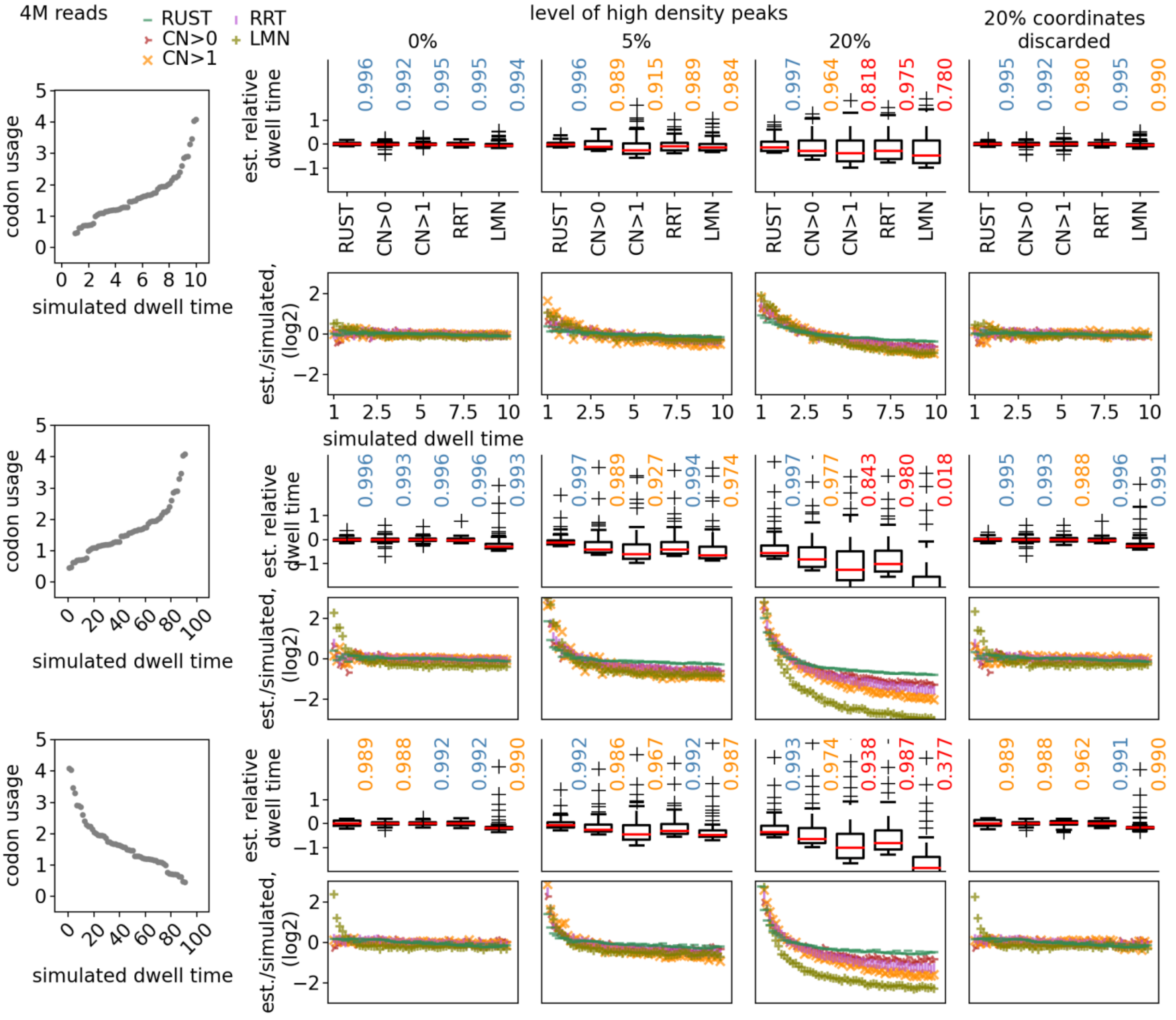

Supplementary Figure 2. Comparison of different normalization approaches

Relationship between the estimated and simulated codon dwell times on a simulated ribosome profiling datasets with five approaches; RUST, CN of all expressed transcripts (CN>0) CN of transcripts with average transcript density >1/nucleotide (CN>1) RRT and LMN. The simulations are shown using three different input parameters; 1) the simulated dwell time correlates positively with codon usage and spans 1 order of magnitude, 2) the dwell time correlates positively with codon usages and spans 2 orders of magnitudes and 3) the dwell time correlates negatively with codon usage and spans 2 orders magnitudes. For each simulation, the distribution of the normalized ratio of the simulated and estimated relative dwell time is displayed (top) as is their coefficient of determination. The estimated/simulated (est./stimulated) relative dwell times of individual codons, ordered from quickest (left) to slowest (right) dwell times, is shown in the bottom subpanel.

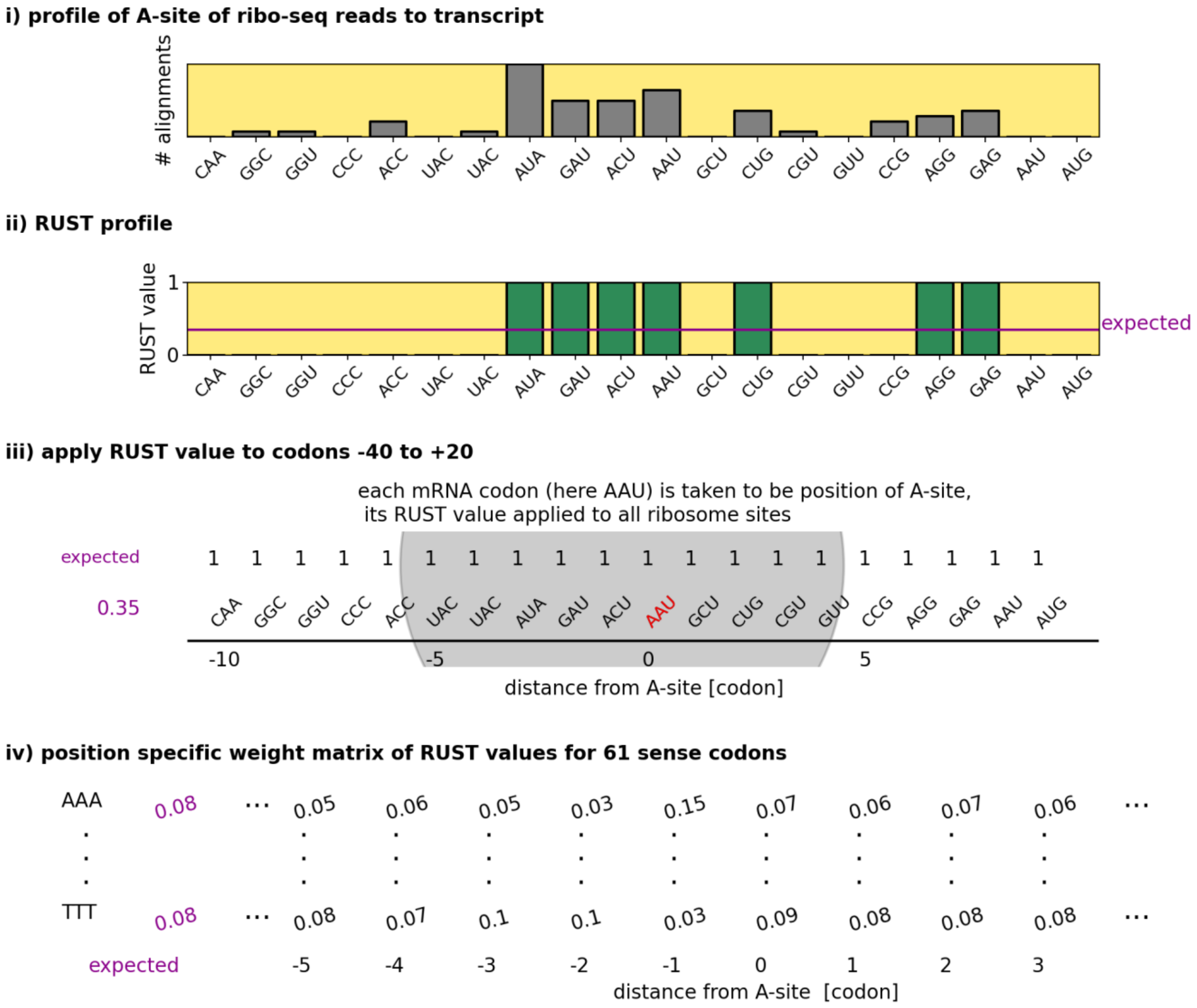

Supplementary Figure 3. A diagram explaining generation of RUST metafootprint profiles for codons. i) ribo-seq (or RNA-seq) reads of fixed length are mapped to each transcript with an offset applied to indicate the position of the A-site codon (Preparatory phase in Supplementary Fig. 1). ii) The profile is converted to a binary profile by the RUST protocol with the average RUST value for the transcript taken to be the expected value (RUST phase in Supplementary Fig. 1). iii) For each individual coding region the observed RUST values are calculated for each location within 60 codon window, from -40 codons to +20 codons. iv) The summative values are obtained for each codon at a position within a window across all windows in all coding regions as well as corresponding average expected RUST values which are not position specific. The observed-to-expected RUST ratio at a specific location indicates whether a particular codon is enriched or depleted. This allows to measure the effect of a codon at a specific location on footprint density.

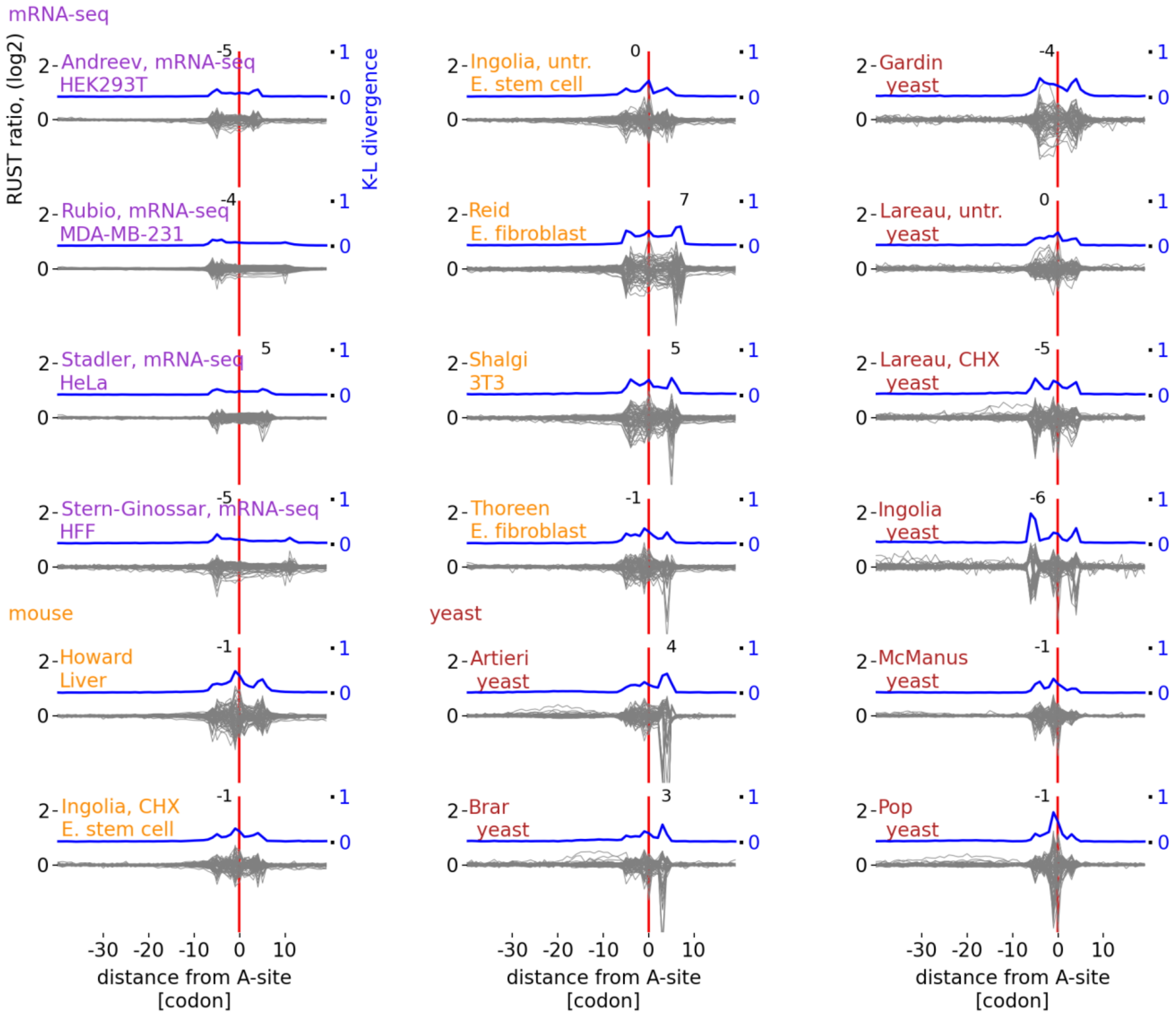

Supplementary Figure 4. Metafootprint profiles of the human mRNA-seq mouse and yeast ribo-seq data. The K-L divergence is shown in blue, the coordinate of K-L maximum is also indicated in each plot. E. used as abbreviation of embryonic.

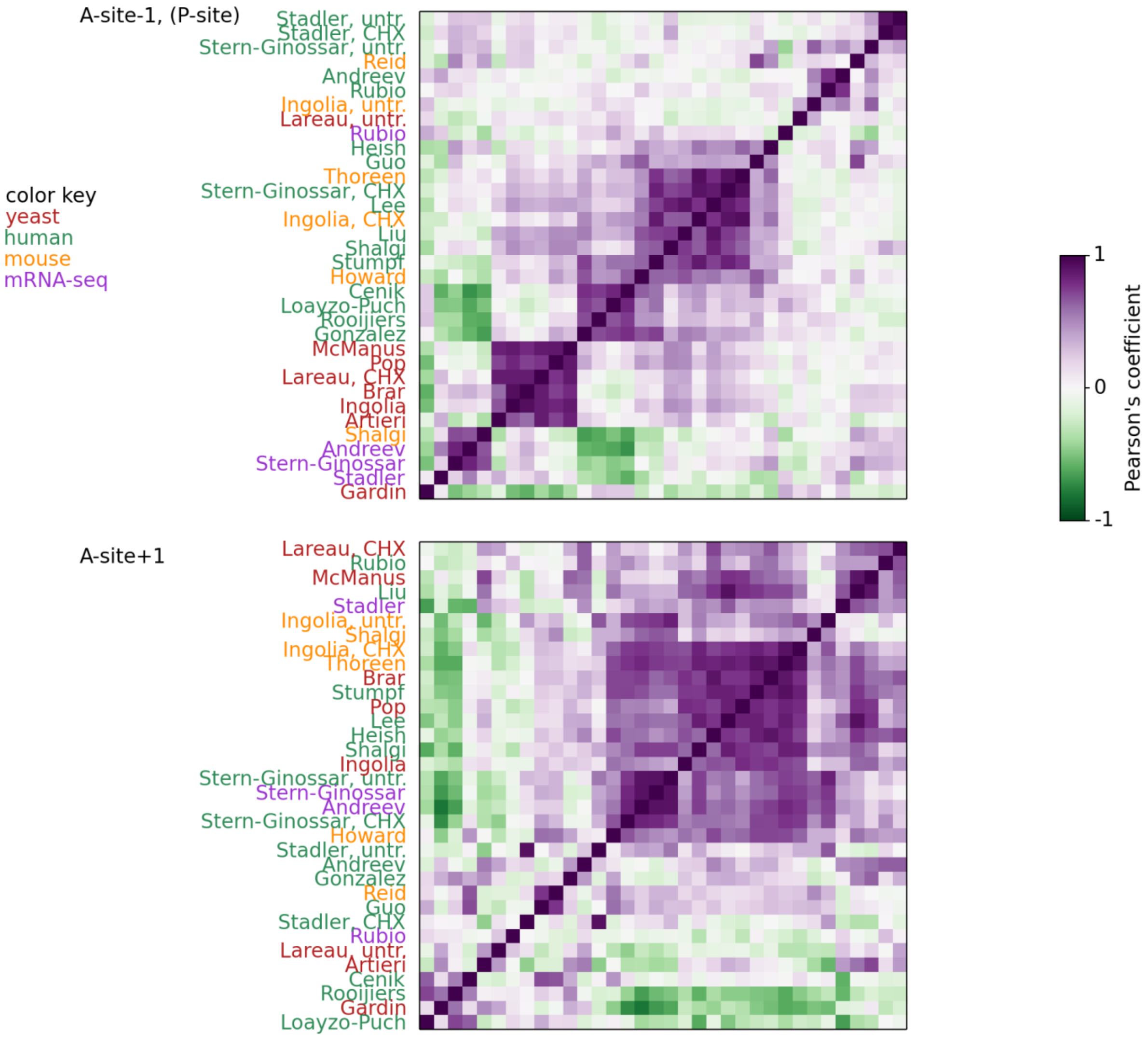

Supplementary Figure 5. Comparison of the datasets using RUST ratios at the codons adjacent to the A-site. Heatmaps produced with the pairwise similarity comparison of codon RUST ratios at the P-site (top) and the site immediately 3’ of the A-site (bottom), as measured by the Pearson’s correlation, for ribo-seq datasets of human (green), yeast (red) and mouse (orange). Also included are human mRNA-seq data (violet). The clustering was created with Scipy using “Euclidean” distance metric with “single” linkage.

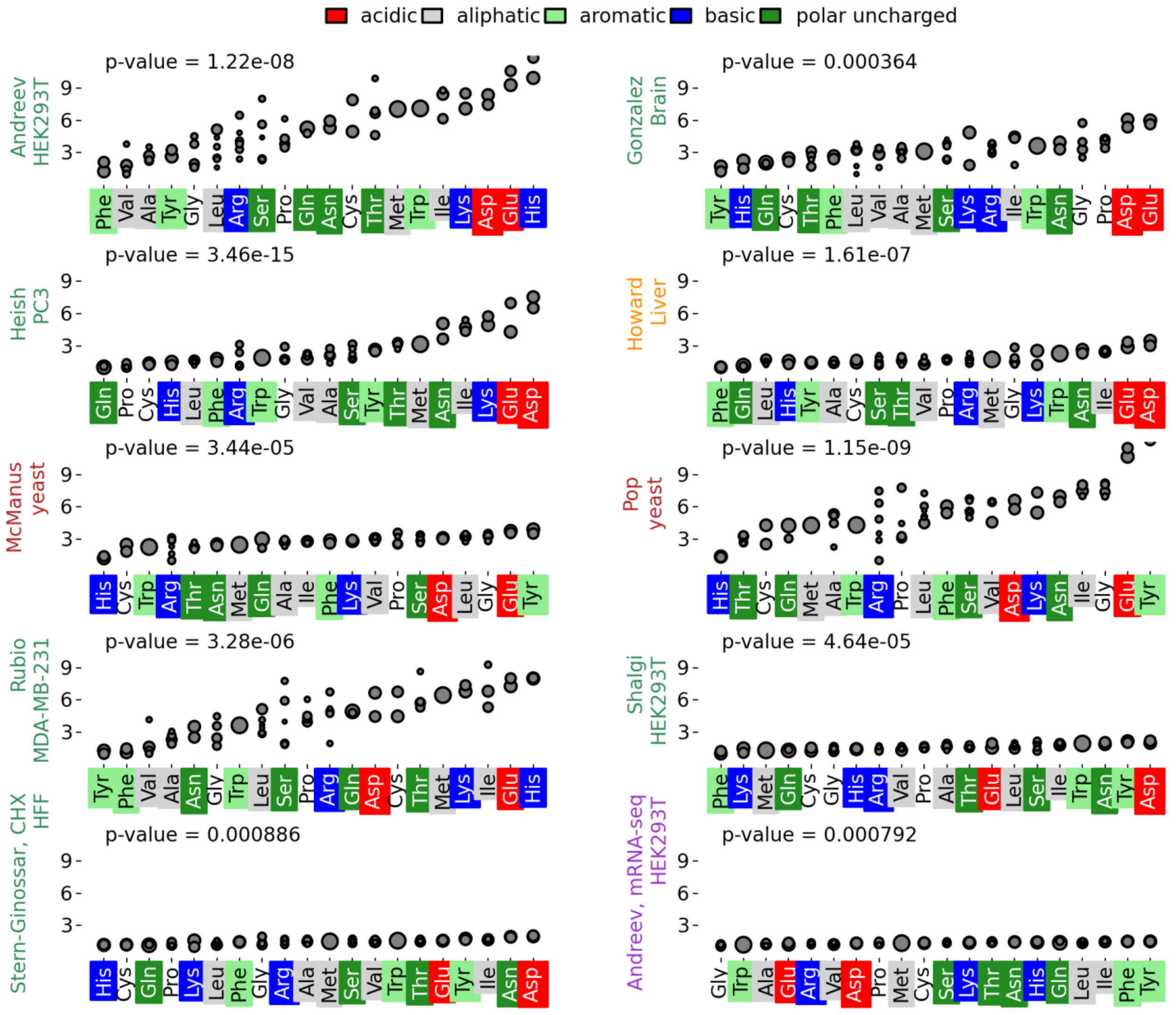

Supplementary Figure 6. RUST ratios for each of 61 codons in the A-site in different datasets

Synonymous codons display lower variation of RUST ratios at the A-site than nonsynonymous codons. The RUST ratios, for 9 ribo-seq datasets and 1 mRNA-seq (bottom right) dataset are scaled relative to the minimum. The 61 sense codons are grouped by the amino acid that they encode. ANOVA was used to calculate the p-values to test whether synonymous codons display lower variation than the background. Note that the low p-value obtained for most of the datasets including the mRNA-seq control, even though that as expected the degree of variation for 61 codons is much lower. The results for all samples available at http://lapti.ucc.ie/rust/

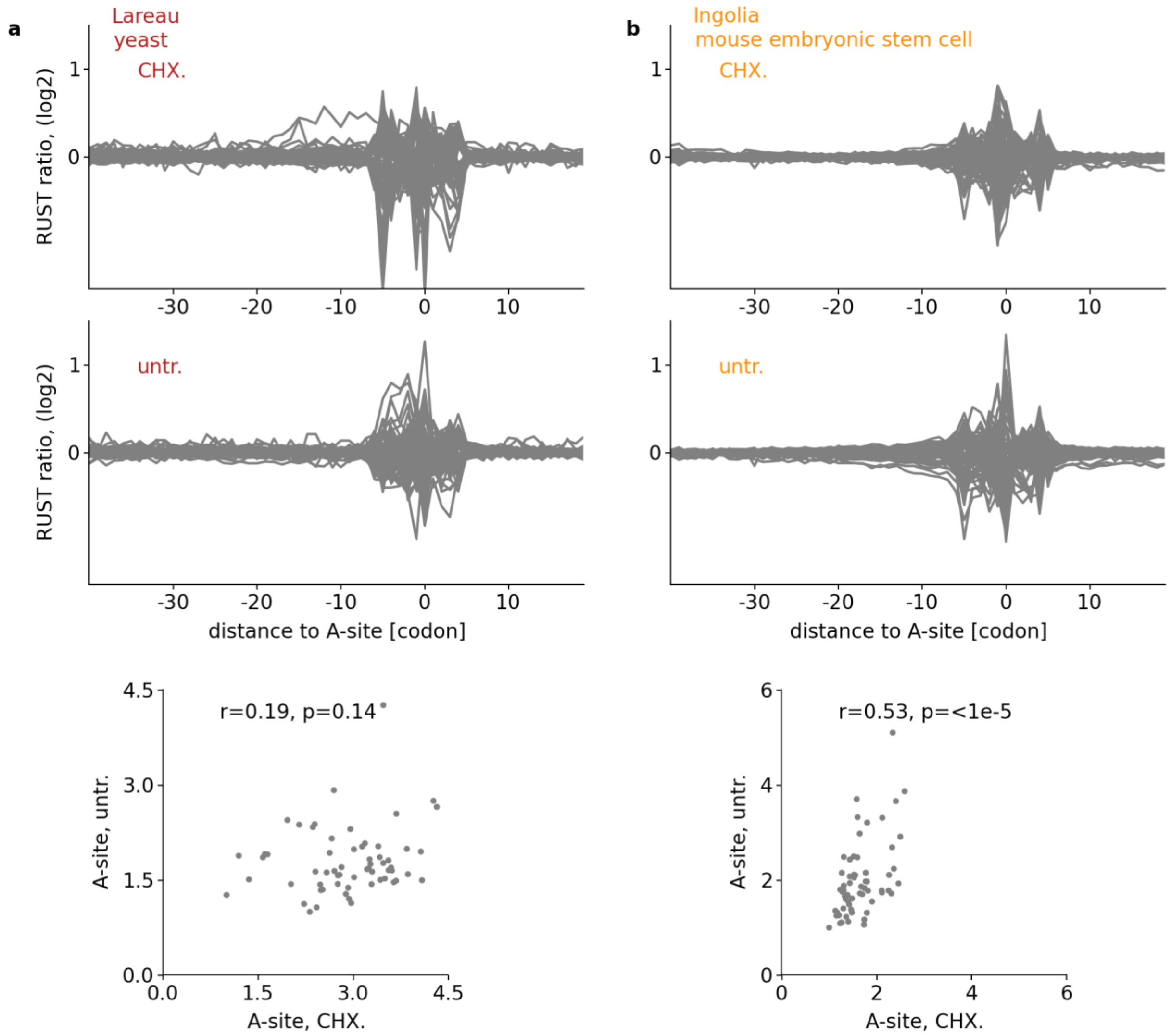

Supplementary Figure 7. The effect of cycloheximide treatment on footprint libraries

Treatment with cycloheximide can strongly influence the frequency of footprints derived from particular codons. (**a**) The metafootprint profile of cycloheximide treated (top left) and untreated (middle left) obtained for yeast data. The comparison of A-site codons RUST ratios for the two datasets is shown at the bottom. (**b**) Similar to (**a**) except for ribo-seq data obtained from mouse cells**.**

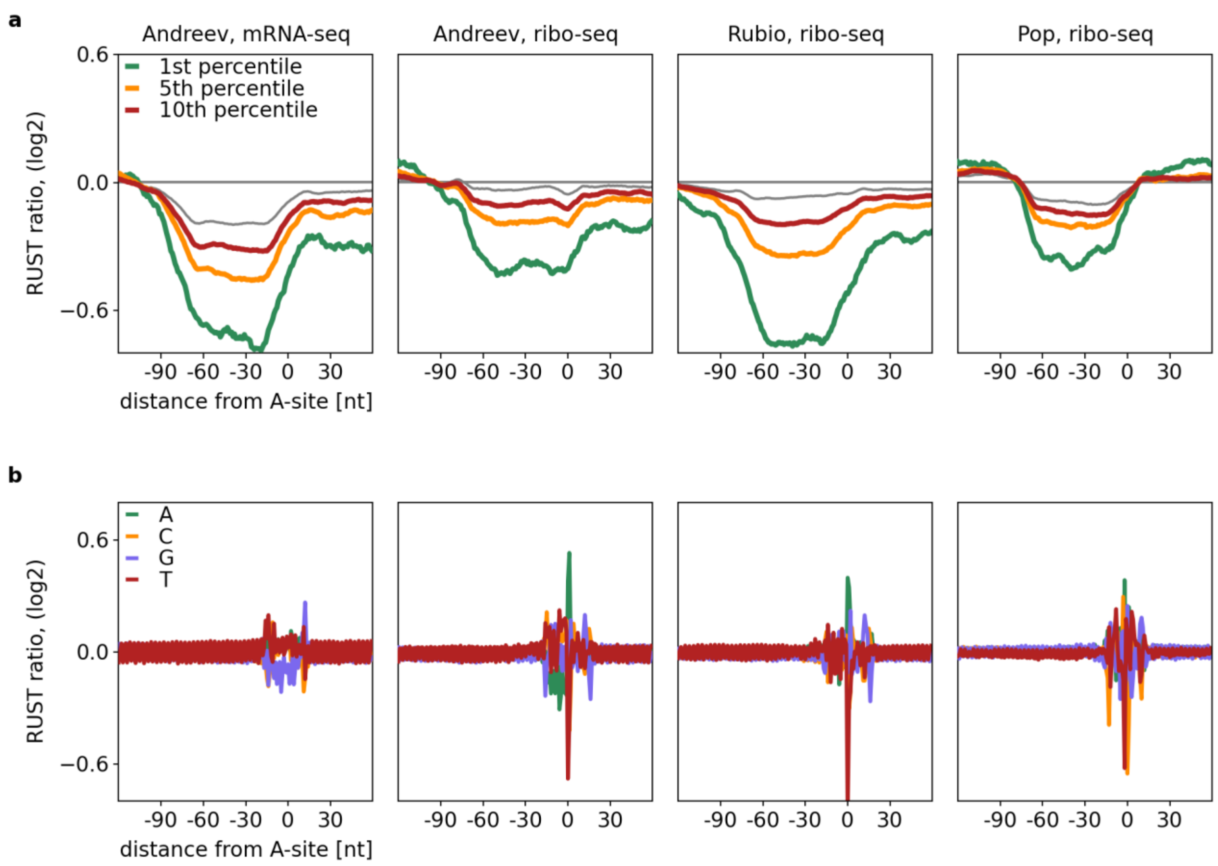

Supplementary Figure 8. Sequence specific factors affecting the composition of ribo-seq libraries. (**a**) The metafootprint analysis for RNA segments potentially forming secondary structures with different stabilities indicated as free energies percentiles. Each point of the line indicates the first nucleotide of an 80 nucleotide window used to calculate the free energy. (**b**) The metafootprint analysis for individual nucleotides. nt used as abbreviation of nucleotide

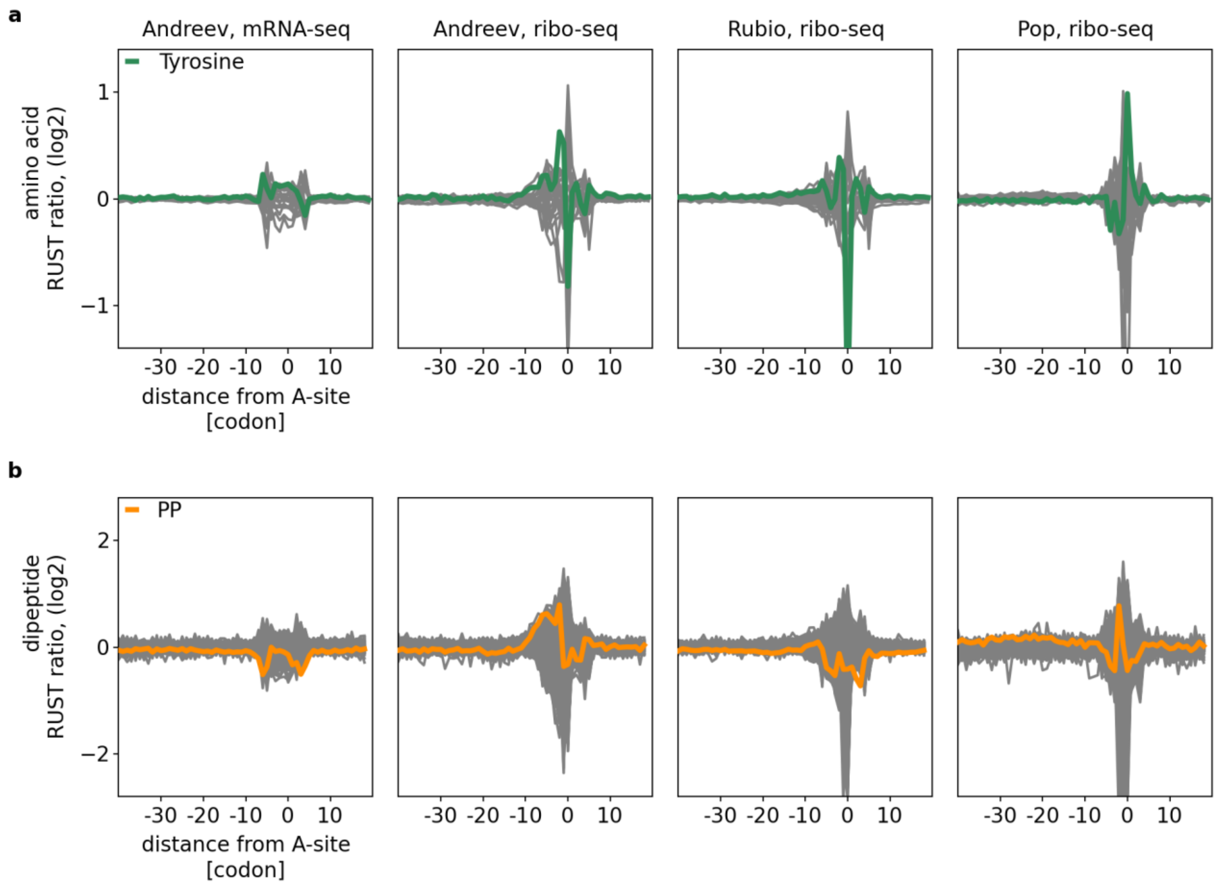

Supplementary Figure 9. RUST metafootprint profiles for amino acids and dipeptides

(**a**) individual amino acids and (**b**) dipeptides show no evidence of influence of nascent peptide region distal from peptidyl transferase center on ribosome footprint density.

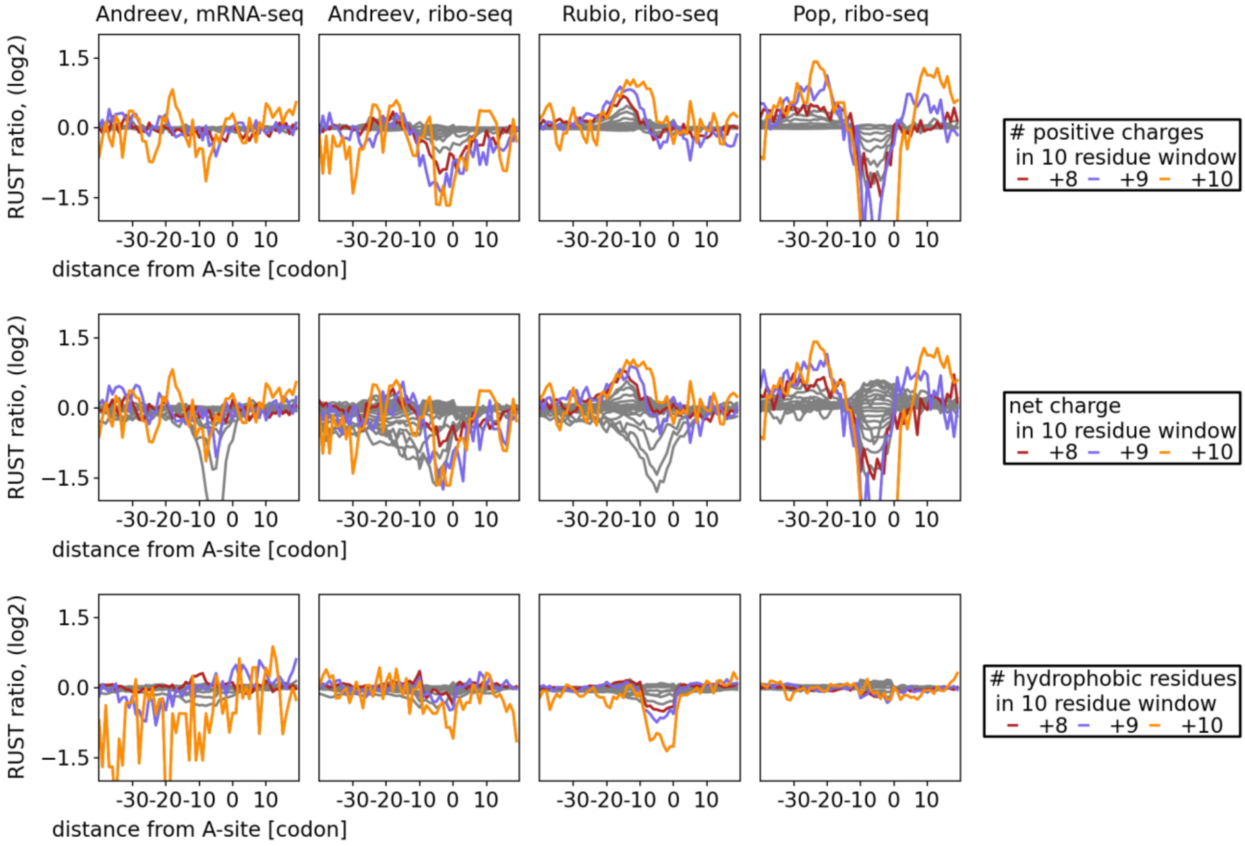

Supplementary Figure 10. RUST metafootprint profiles for of physiochemical properties of encoded peptides. The physicochemical properties of the peptides were measured with a 10 codon sliding window (at 1 codon step size). The properties are classified by the number of positively charged amino acids, the net charge and the number of hydrophobic residues within each window. The RUST ratios for 10-codon windows are plotted at the position of the most N-terminal residue in the window. nt used as abbreviation of nucleotide

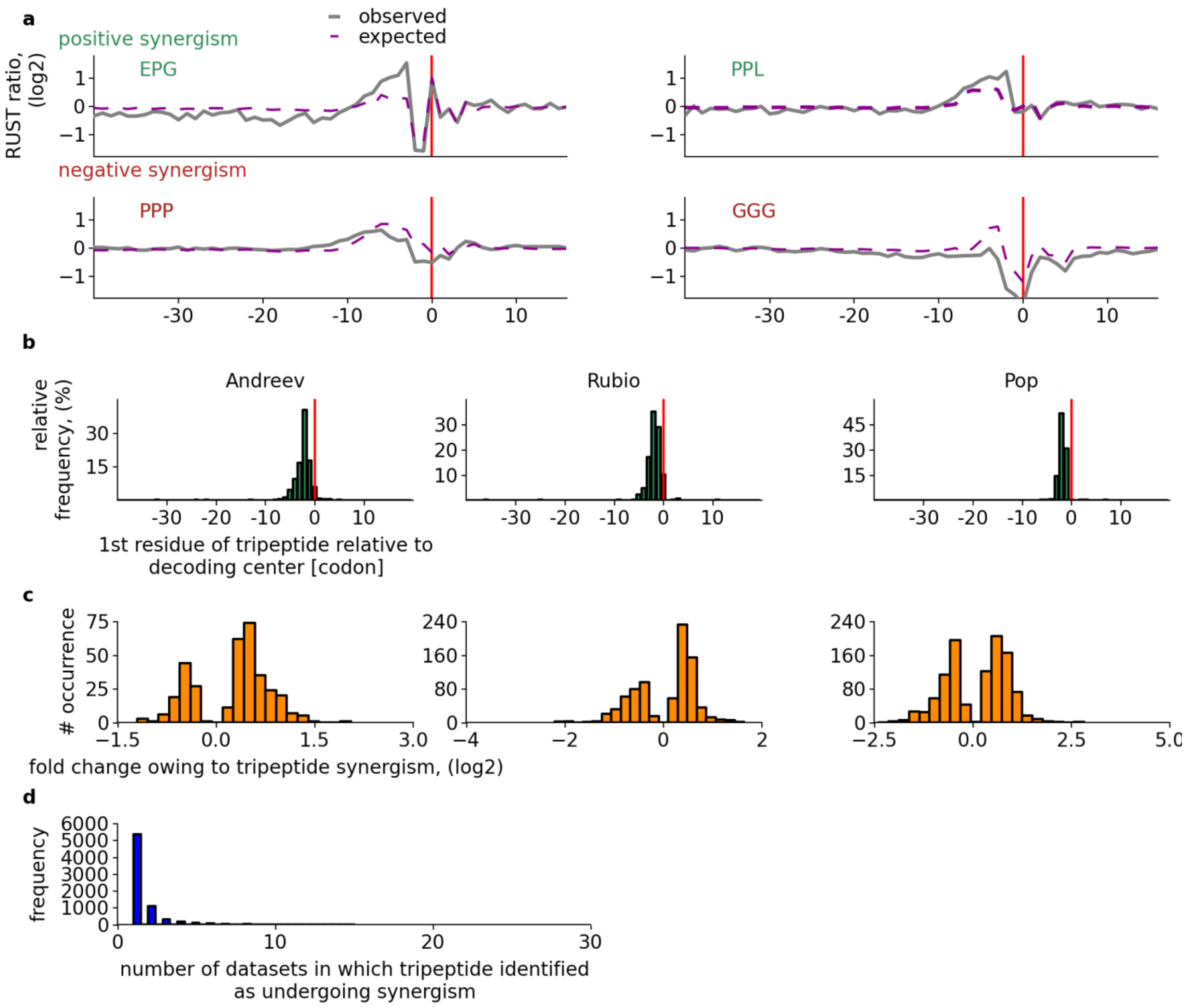

Supplementary Figure 11. Synergism of adjacent codons have minor influence on ribo-seq read density. (**a**) Examples where a significant difference exists between the observed (grey) and expected (purple) RUST ratio for tripeptides. The expected RUST ratio is estimated by the combined effects of individual amino acids. (**b**) The relative frequencies of synergistic interactions with tripeptides detected across different positions of the ribosome. Stringent standard score 4 (see Methods) was used for identifying such cases. The positions of the N-terminal residues are indicated. (**c**) The fold change between the expected-to-observed RUST ratios for cases of synergistic interactions with tripeptides. Motifs with a $\log_2$ change close to 0 were not included as they did not pass the standard score threshold. (**d**) Distribution of synergetic tripeptides detected in 30 datasets. Most of synergetic tripeptides are specific to a single dataset.

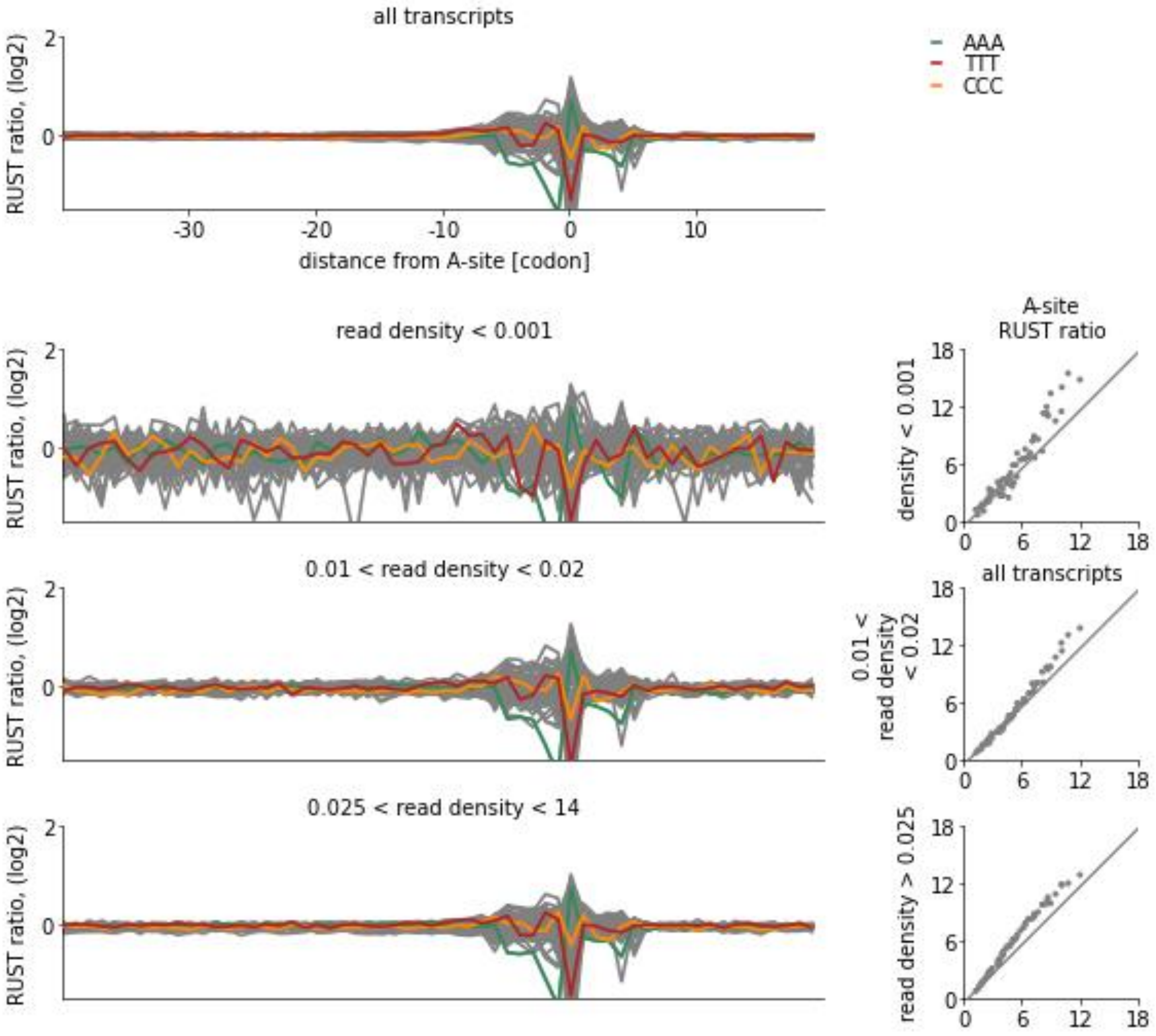

Supplementary Figure 12. The effect of sequencing coverage and expression levels

RUST profile produced with a subsets of genes grouped by the averaged footprint density (indicated). The RUST metafootprint profile for the entire dataset (top) followed by other metafootprint profiles each produced with ~10% of the total number of expressed genes. The correlations between the A-site codon RUST ratios from the total dataset and its subsets are displayed on the right.

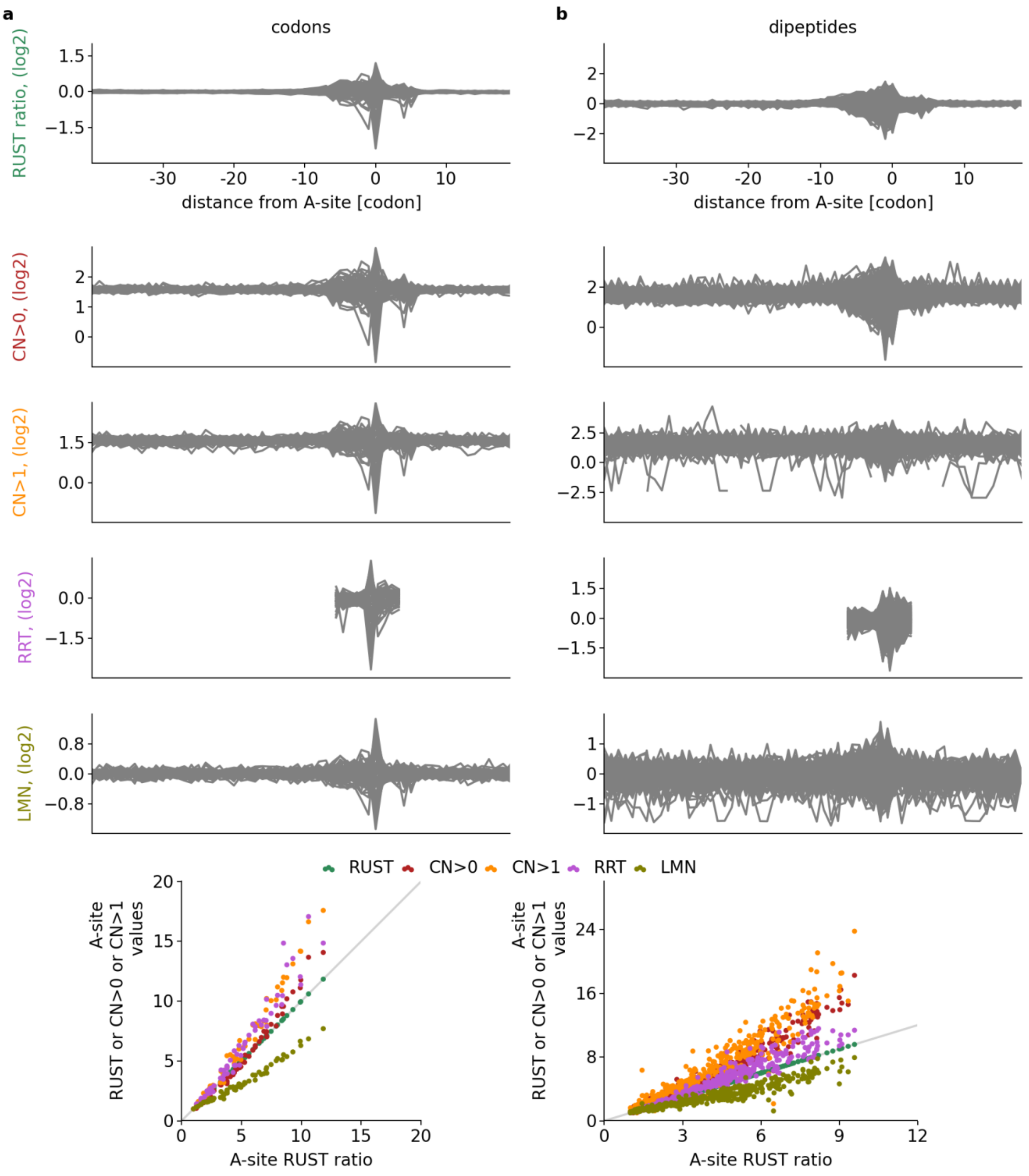

Supplementary Figure 13. RUST, CN, RRT and LMN metafootprint profiles obtained with real data. (**a**) The metafootprint profiles for A-site codons obtained using different normalization techniques. The correlation of the decoding center between RUST and other methods is shown at the bottom. (**b**) The metafootprint profiles of dipeptides. Both panels show that RUST metafootprint profiles have a higher signal to noise ratio.

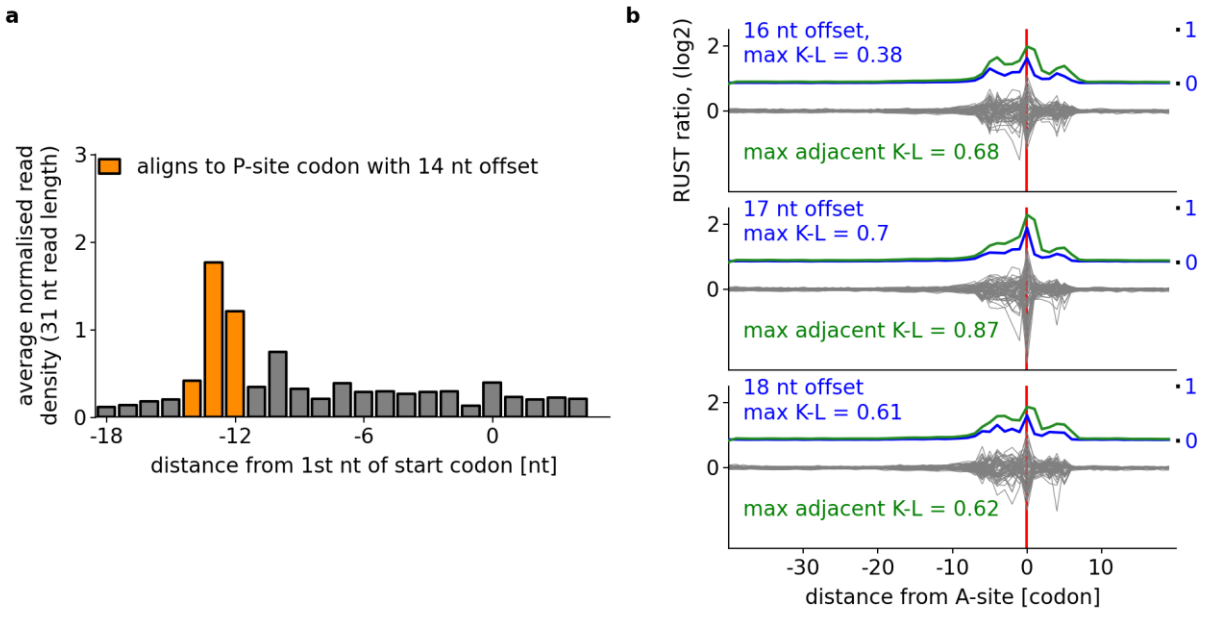

Supplementary Figure 14. Identifying offset to the A-site

(**a**) Metagene profile at the initiation site for 31 nucleotides long footprints. A 14 nucleotide offset from the 5' end (orange) is the most likely distance to the P-site. (**b**) Metafootprint profiles of RUST ratios obtained with various offsets. The K-L divergence (blue line) is greatest at the A-site using a 17 nucleotide offset. In situations where the P-site has large influence on decoding rate the K-L divergence for two adjacent codons (green) is a more accurate approach for identifying correct offset to the decoding center (P- and A-sites). The data used for this figure are from "Andreev" dataset. nt used as abbreviation of nucleotide

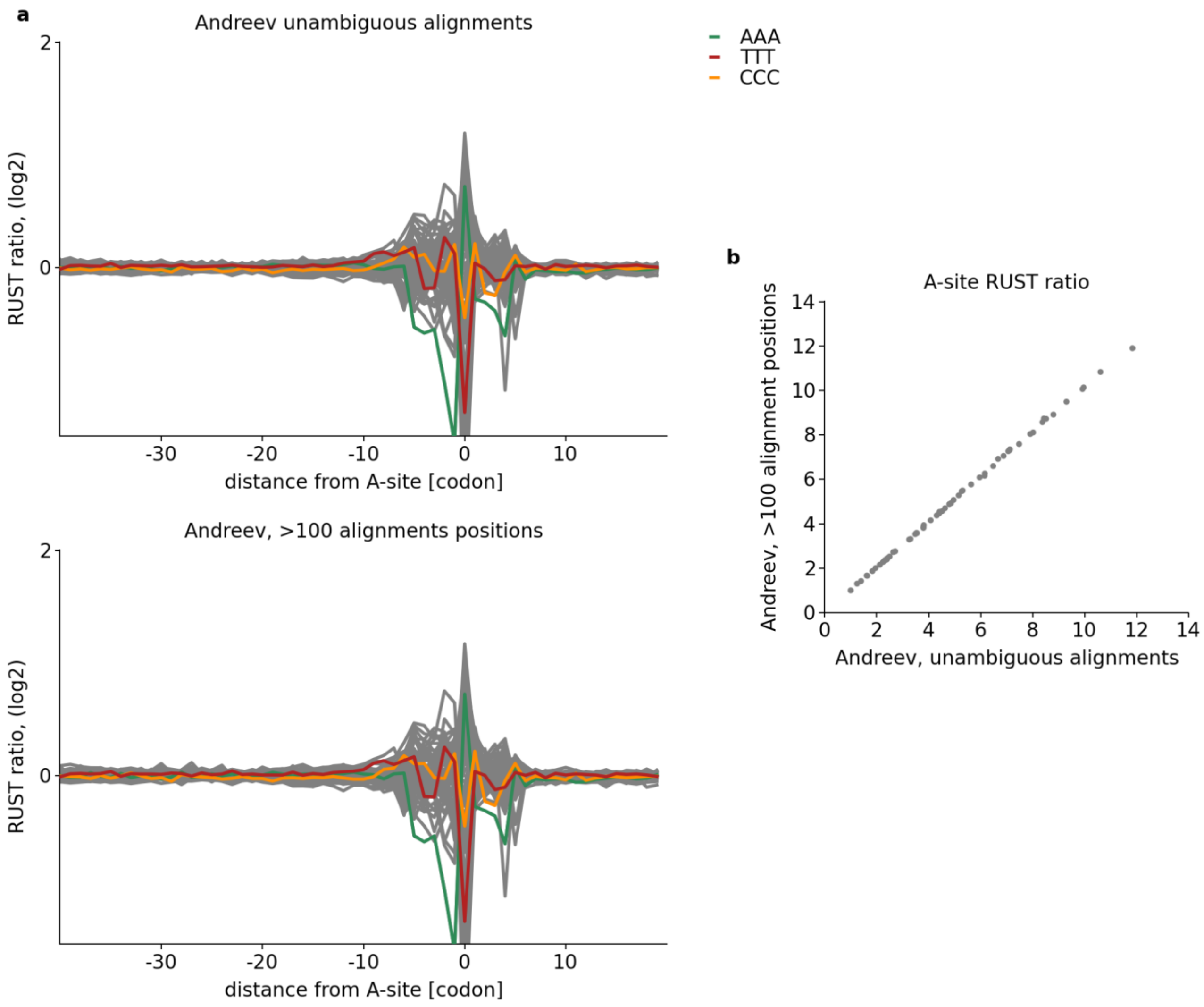

Supplementary Figure 15. The effect of ambiguous reads on RUST metafootprint profiles

(**a**) The metafootprint profiles obtained from unambiguous reads only (top left) or including reads that align to less than 100 positions (bottom left) are nearly identical. (**b**) The comparison of A-site codon RUST ratios inferred from datasets with and without ambiguous reads inclusion.

	Description	PubMed Id	SRA file(s)	Cell line/Tissue	Aligned reads	Read length	A-site offset	Reads used to produce profile
**Human**								
	Andreev	25621764	SRR1173909,SRI	HEK293T	9678014	31	17	1418111
	Cenik	26297486	SRR1803149	EBV-transformed lymphoblastoid	4300083	30	15	228628
	Gonzalez	25122893	SRR1562539	Brain	12349880	29	16	1679952
	Guo	20703300	SRR057512	HeLa	8613493	30	14	1277108
	Heish	22367541	SRR403883	PC3	4074203	29	17	530051
	Lee	22927429	SRR618771	HEK293	10695143	29	16	1042369
	Liu	23290916	SRR619083	HEK293	13579417	29	16	1171199
	Loayzo-Puch	23594524	SRR627620	BJ fibroblast	13429315	29	15	1940357
	Rooijiers	24301020	SRR935448	BJ fibroblast	16613559	29	16	569247
	Rubio	25273840	SRR1573934	MDA-MB-231	61819405	32	17	6470387
	Shalgi	23290915	SRR648667	HEK293T	8796151	32	17	945078
	Stadler, CHX	22045228	SRR407637	HeLa	11956304	32	17	1870693
	Stadler, untr.	22045228	SRR407643	HeLa	12555437	32	17	977131
	Stern-Ginossar	23180859	SRR609197	human foreskin fibroblasts	7641866	30	15	1979525
	Stern-Ginossar	23180859	SRR592961	human foreskin fibroblasts	11228476	31	17	900948
	Stumpf	24120665	SRR970561	HeLa	23870096	31	17	1183436
**Mouse**								
	Howard	23696641	SRR826795	Liver	40385932	34	17	5001378
	Ingolia, CHX	22056041	SRR315601	Embryonic stem cell	4721388	31	17	447215
	Ingolia, untr.	22056041	SRR315616	Embryonic stem cell	6146735	31	17	680077
	Reid	25215492	SRR1066893	Embryonic fibroblast	5832921	33	14	853619
	Shalgi	23290915	SRR649752	3T3	2685761	30	14	477120
	Thoreen	22552098	SRR449467	Embryonic fibroblast	4053303	30	16	483481
**Yeast**								
	Artieri	25294246	SRR1049093		38082586	28	15	7866955
	Brar	22194413	SRR387871		8429595	28	17	1288175
	Gardin	25347064	SRR1506632		8912388	29	14	1025309
	Ingolia	19213877	SRR014374,SRR014375, SRR014376		1264121	29	17	271584
	Lareau, CHX	24842990	SRR1363415,SRR1363416		8007112	28	16	2321224
	Lareau, untr.	24842990	SRR1363412,SRR1363413, SRR1363414		11249812	29	17	2393769
	McManus	24318730	SRR948555		13632476	29	17	3089629
	Pop	25538139	SRR1688547		9676033	29	17	2321278
**Human, mRNA-seq**								
	Andreev	25621764	SRR1173911, SR	HEK293T	20165353	27	17	903043
	Rubio	25273840	SRR1573935	MDA-MB-231	84575026	50	17	11802212
	Stadler	22045228	SRR407636	Hela	10031834	36	17	789626
	Stern-Ginossar	23180859	SRR592963	human foreskin fibroblasts	10274713	50	17	260792
